# The effect of immobilisation strategies on the ability of peptoids to reduce the adhesion of *P. aeruginosa* strains to contact lenses

**DOI:** 10.1016/j.exer.2024.110149

**Published:** 2024-11-20

**Authors:** Manjulatha Sara, Sudip Chakraborty, Renxun Chen, Dennis Palms, Georgio Katsifis, Zhongyan Li, Syamak Farajikhah, Vinod Massedupally, Alex Hui, Edgar H.H. Wong, Naresh Kumar, Krasimir Vasilev, David Mackenzie, Linda Losurdo, Farida Dehghani, Havard Jenssen, Kristian Sorensen, Jennifer S. Lin, Annelise E. Barron, Mark Willcox

**Affiliations:** a School of Optometry and Vision Science, UNSW Sydney, Australia; b School of Chemistry, UNSW Sydney, Australia; c Biomedical Nanoengineering Laboratory, College of Medicine and Public Health, Flinders University, Bedford Park, SA, 5042, Australia; d School of Physics, University of Sydney, NSW, 2006, Australia; e University of Sydney, Australia; f Centre for Ocular Research and Education, University of Waterloo, Canada; g School of Chemical Engineering, UNSW Sydney, Australia; h Department of Science and Environment, Roskilde University, 4000, Roskilde, Denmark; i Department of Bioengineering, School of Medicine & School of Engineering, Standford University, California, 94305, USA

**Keywords:** Contact lens, Microbial keratitis, Antimicrobial peptoids, Covalent attachment, *P. aeruginosa*

## Abstract

**Aim:**

Previous studies have demonstrated that contact lenses coated with the antimicrobial cationic peptide Mel4, a derivative of melimine, can reduce the occurrence of keratitis. However, the antimicrobial activity of Mel4 weakened over time due to its susceptibility to proteolytic degradation. Oligo-*N*-substituted glycine peptoids such as TM5 and TM18 possess antimicrobial properties and are resistant to proteolytic breakdown. This study focused on exploring methods for covalently attaching these peptoids to contact lenses to enhance their durability and performance *in vitro*.

**Methods:**

The peptoids TM5 and TM18 were covalently attached to etafilcon lenses via carbodiimide chemistry (EDC/NHS), oxazoline plasma, and plasma ion immersion implantation (PIII). The lenses were analysed using X-ray photoelectron spectroscopy (XPS), surface charge, and hydrophobicity. Inhibition of adhesion of multidrug-resistant *Pseudomonas aeruginosa* and cytotoxicity on corneal epithelial cells were evaluated. The impact of moist heat sterilization on activity was also assessed.

**Results:**

XPS confirmed peptoid binding to lenses. Peptoid coatings slightly increased contact angles (≤23°) without affecting overall charge. Peptoids, bound via carbodiimide, inhibited *P. aeruginosa* adhesion by over 5 log10 CFU per lens, outperforming melimine, which required six times the concentration for a 3 log10 reduction. Peptoids attached via oxazoline or PIII reduced adhesion by > 5 log10 CFU. All covalent methods significantly reduced bacterial adhesion compared to untreated lenses (*P* < 0.0001). Peptoid-bound lenses were non-toxic to corneal epithelial cells. Sterilization did not affect carbodiimide-treated lenses but reduced the activity of oxazoline and PIII surfaces by 1–2 log10 CFU.

**Conclusion:**

Peptoids TM5 and TM18 effectively reduced *P. aeruginosa* adhesion on lenses, with carbodiimidebound surfaces retaining activity post-sterilization, showing promise for the development of antimicrobial contact lenses.

## 1. Introduction

Contact lenses are commonly worn to correct refractive errors, to manage diseases of the cornea such as keratoconus or, more recently, to reduce the growth of the eye and the consequent myopia (short-sightedness) (Gonzalez-Meijome et al., 2016; Morgan et al., 2021; Schaeffel et al., 1988). Most contact lens wearers wear soft lenses such as hydrogels or silicone hydrogels (Morgan et al., 2024). However, contact lens wear comes with the risk of developing infection or inflammation of the ocular surface. Infection rates range from approximately 2 to 20 per 10,000 wearers per year (Stapleton, 2020). Inflammation is more common and occurs at 2670 per 10,000 wearers (Kalaiselvan et al., 2021), or 42 per 10,000 patient-years (Bullimore and Richdale, 2023).

Both infection and inflammation (which can be collectively referred to as corneal infiltrative events; CIEs) can be caused by microbial contamination of contact lenses (Kalaiselvan et al., 2022; Szczotka--Flynn et al., 2009). The most common bacteria associated with infection of the cornea, called microbial keratitis, during contact lens wear is *Pseudomonas aeruginosa* (Liu et al., 2019; Stapleton and Carnt, 2012; Subedi et al., 2018). This bacterium and other gram-negative bacteria also commonly cause non-infectious inflammation during contact lens wear (Holden et al., 1996; Willcox et al., 2011).

To mitigate the occurrence of CIEs, antimicrobial contact lenses have been explored. Various approaches have been investigated such as the addition of silver or other metal ions to lenses or adding antimicrobial cationic peptides (Dutta et al., 2016; Khan et al., 2021; Ma et al., 2022). One strategy that covalently attached the cationic antimicrobial peptide (AMP) Mel4 to lenses using *N*-ethyl-*N*-(3-(dimethyl amino) propyl) carbodiimide/n-hydroxy succinimide (EDC/NHS) chemistry has progressed to clinical trials (Dutta et al., 2014). Mel4-coated lenses, and lenses coated with its parent AMP melimine, showed potent efficacy against antibiotic susceptible and multidrug-resistant *P. aeruginosa* and *S. aureus* strains, other gram-negative and gram-positive bacteria, the fungi *Candida albicans* and *Fusarium solani*, and Acanthamoeba (Dutta et al., 2013, 2017, 2018). In human studies, the Mel4-coated lenses were safe and comfortable to wear (Dutta et al., 2014), did not alter the most common microbes cultured from eyes during wear (Kalaiselvan et al., 2022), and reduced the incidence of CIEs by 69% when worn for 14 days and nights compared to control uncoated lenses (Kalaiselvan et al., 2021). However, Mel4-coated lenses lost antimicrobial activity compared to untreated controls after six nights of wear, most likely due to the degradation of the peptide by tear proteases (Kalaiselvan et al., 2023). If antimicrobial contact lenses can maintain their efficacy, this could enhance their capacity to minimize CIEs.

Peptoids are mimics of AMPs. They can be synthesized with the side chain attached to the backbone nitrogen instead of the α-carbon and, as such, are resistant to the action of proteolytic enzymes (Chongsiriwatana et al., 2011; Sara et al., 2024). The incorporation of alkyl substituents and one or two *N*-substituted glycine residues (Sara et al., 2024), and variations in the lengths of carbon chains impart diverse and modifiable characteristics (Chongsiriwatana et al., 2008). This versatility enables them to emulate the actions of natural AMPs, making them effective antimicrobial agents (Chongsiriwatana et al., 2011). Peptoids can have activity at significantly lower minimum inhibitory (2–32-fold) concentrations compared to AMPs (Molchanova et al., 2020). They are also effective when attached to surfaces. For example, a peptoid consisting of a linear poly(*N*-methoxyethyl glycine), when bound on titanium oxide surfaces, reduced adhesion of *Escherichia coli* and *Staphylococcus aureus* by 95% (Lau et al., 2012). Additionally, poly(*N*-methyl glycine)/polysarcosine peptoid brushes significantly reduced the adhesion of *P. aeruginosa* for over 7 weeks in laboratory experiments (Lau et al., 2012). In previous studies TM5 and TM18 emerged from the panel of peptoids as having a wide therapeutic index, indicating their strong potential for therapeutic applications (Sara et al., 2024). These attributes suggest peptoids may be an excellent alternative to AMPs for producing long lasting antimicrobial contact lenses. Therefore, the present study investigated the feasibility of immobilizing peptoids onto contact lenses and evaluating their properties.

## 2. Materials and methods

### 2.1. Compound synthesis

**Fig. 1.**
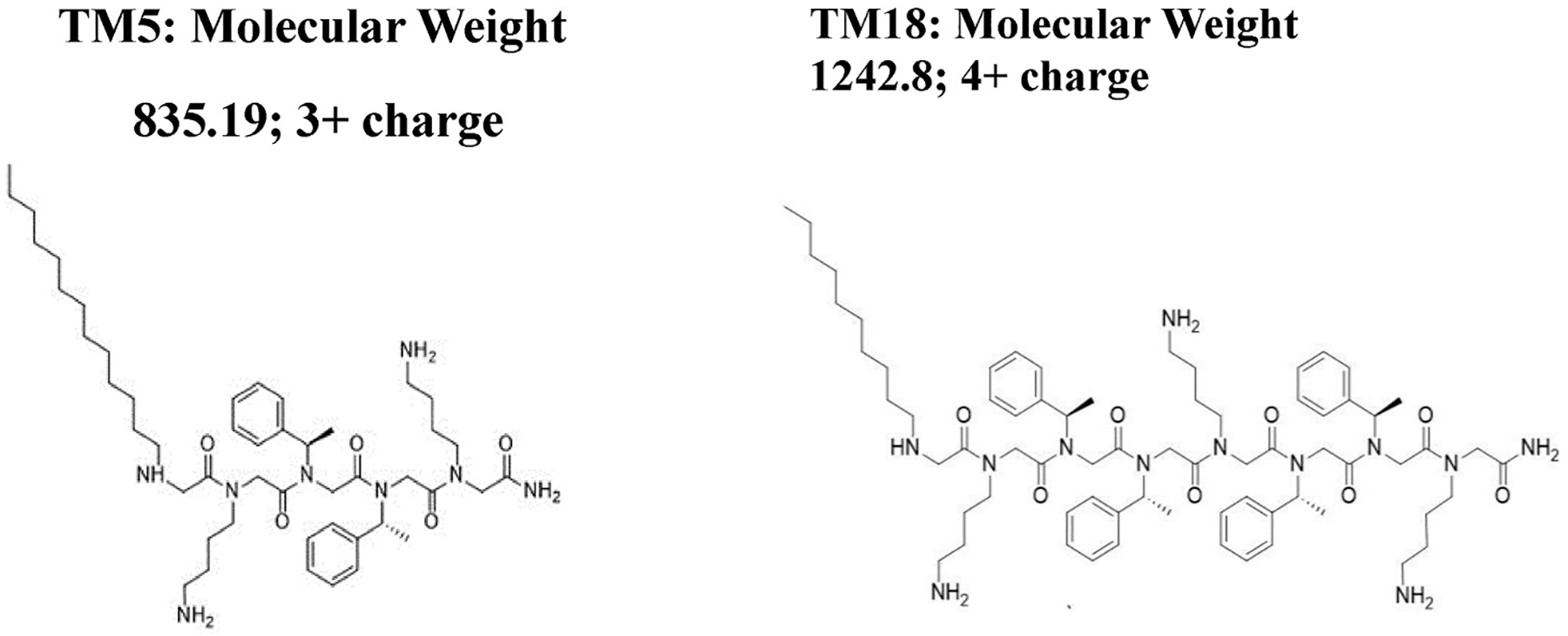
Chemical structures of peptoids TM5 and TM18.

The cationic peptide melimine was synthesized through solid-phase peptide synthesis as described previously and produced at ≥90% purity (Yasir et al., 2019). Peptoids TM5 and TM18 (Fig. 1), previously shown to have excellent antimicrobial activity against ocular isolates of *P. aeruginosa*, with minimum inhibitory concentrations of 7.8–31 μg mL^−1^ (Sara et al., 2024), were synthesized using the submonomer method with synthetic amines (Connolly et al., 2021). *N*-substituted amines were directly coupled to a solid amide resin, and new moieties were introduced step by step. *N* -alkylated amines were added by reacting primary amines with bromoacetic acid. Once the desired sequence was achieved, amines were deprotected, and the peptoids were cleaved from the solid support using trifluoracetic acid. High-performance liquid chromatography demonstrated that they were ≥97% pure (Chongsiriwatana et al., 2011; Patch and Barron, 2003).

### 2.2. Bacterial strains

*P. aeruginosa* 6294 (an *exoS* positive invasive strain, isolated from microbial keratitis), *P. aeruginosa* 123 (an *exoU* positive cytotoxic strain, isolated from microbial keratitis) and *P. aeruginosa* ATCC 19660 (an *exoU* positive strain cytotoxic strain, isolated from a case of septicaemia) were used in this study (Kulasekara et al., 2006; Subedi et al., 2020; Zhu et al., 2006).

### 2.3. Contact lenses

Etafilcon A contact lenses (Johnson & Johnson Vision Care Inc., Jacksonville, FL, USA), which has previously been used for Mel4-coatings that progressed to a Phase III clinical trial (Kalaiselvan et al., 2021), were removed from their packs, transferred into 24 well polystyrene plates (Greiner CELLSTAR; Greiner Bio-One, Frickenhausen, Germany) and washed three times in 2 mL sterile phosphate-buffered saline (PBS; NaCl 8 g/L, KCl 0.2 g/L, Na_2_HPO_4_ 1.4 g/L, KH_2_PO_4_ 1.4 g/L; pH 7.4). The lenses were then either used as controls or activated to allow covalent attachment of the antimicrobial agents.

### 2.4. Activation of contact lenses

Three different attachment strategies were used, carbodiimide chemistry, oxazoline plasma deposition and plasma immersion ion implantation. The protocol used for binding the compounds via carbodiimide chemistry has been previously described (Dutta et al., 2013). Briefly, lenses were washed with sodium acetate buffer (pH 5.0), followed by immersion in a solution containing 1-ethyl-3-(3-dimethylaminopropyl carbodiimide hydrochloride (EDC) and *N*-hydroxy succinimide (NHS), then incubated at room temperature for 15–30 min with the peptoids or melimine.

The method for deposition of the oxazoline plasma has been previously published (MacGregor et al., 2019). Lenses were washed three times in Milli-Q water and then dried under nitrogen. A capacitively coupled plasma reactor operating at 56 mHz was used to deposit thin films of 2-methyl-2-oxazoline. After placing the lenses in the reactor chamber, the system was brought to base pressure (2 × 10^−2^ mbar). Then, an air plasma cleaning was carried out for 3 min at pressure of 1 × 10^−1^ mbar and using power of 50 W. The chamber what then again brought to base pressure. Deposition of 2-methyl-2-oxazoline was then carried out at working pressure of 2 × 10^−1^ mbar, corresponding to a flow rate of 8 standard cubic centimetres^3^/minute, and power of 50 W for 3 min. The lenses were then flipped over, and the process was repeated to ensure that coatings containing reactive oxazoline rings were deposited on both sides of the lenses. Under these conditions, a film thickness of 50 nm is deposited (MacGregor et al., 2019; Macgregor-Ramiasa et al., 2015; Ramiasa et al., 2015).

The attachment strategy for plasma immersion ion implantation (PIII) using nitrogen gas has been previously published (Tran et al., 2019a). Briefly, after washing in Milli-Q water, contact lenses were air dried and then placed in a borosilicate conical Erlenmeyer flask connected to a RUP6 pulse generator (GBS Electronik GmbH, Dresden, Germany). Nitrogen gas was introduced into the flask at a pressure of 350 mTorr. A pulsed DC dielectric barrier discharge was created in the flask with an application of negative polarity pulses of 7 kV to an external copper electrode. The pulses were applied at a frequency of 1500 Hz and a pulse length of 40 μs for a duration of 20 min (Kruse et al., 2021; Tran et al., 2019a). This treatment causes bond breakages and generates persistent carbon-centred radicals in a modified substrate layer. The long-lived radicals can diffuse to the surface where they bind biomolecules that are already physically adsorbed to form covalent bonds. Lenses were flipped over after one side bad been treated, and the procedure was repeated to ensure that both sides of the lenses were treated with PIII.

### 2.5. Attachment of compounds to activated lenses

For melimine, after activation by carbodiimide, lenses were washed in PBS then melimine was added at a concentration of 3 mg mL^−1^, as this had been previously shown to produce optimal antimicrobial activity (Dutta et al., 2016). For oxazoline surfaces, melimine was evaluated at soaking concentrations of 500 μg mL^−1^, 1 mg mL^−1^, 2 mg mL^−1^ and 3 mg mL^−1^. In the case of PIII-treated surfaces, melimine was applied at soaking concentrations of 1 mg mL^−1^, 2 mg mL^−1^ and 3 mg mL^−1^ (500 μg mL^−1^ was excluded due to its suboptimal activity with oxazoline plasma). Peptoids TM5 and TM18 were added in concentrations of 100, 250, and 500 μg mL^−1^ to carbodiimide-activated lenses to determine their optimal concentration for antibacterial inhibition activity. After determination of the optimum soaking concentration for carbodiimide surfaces, peptoids were applied at a fixed concentration of 500 μg mL^−1^ for both oxazoline and PIII-activated surfaces. Contact lenses were incubated for 2 h in 2 mL of 10% wt/vol sodium chloride (Sigma Aldrich, Burlington, Massachusetts, US), then washed three times in sterile PBS before being exposed to bacteria. All lenses were produced in at least two batches.

### 2.6. Antibacterial inhibition activity of lenses

Initially, to determine the concentrations of melimine, TM5 and TM18 for optimum activity, experiments were conducted with strain ATCC 19660 on carbodiimide surfaces only. Subsequent adhesion studies used all three strains. Previously published methods were followed (Dutta et al., 2016; Kalaiselvan et al., 2021), with slight modifications. Briefly, *P. aeruginosa* strains were grown in tryptone soya broth (Oxoid, Basingstoke, UK) overnight at 37 °C. After washing cells in PBS, they were resuspended in PBS to an optical density at 660_nm_ of 0.1 (equivalent to 10^8^ colony forming units per millimetre; CFU mL^−1^). After further dilution to 10^6^ CFU mL^−1^, 1 mL was dispensed into wells of 24 well polystyrene plates (Greiner Bio-One). Control uncoated and coated lenses were then placed into the wells and incubated at 37 °C for 18 h with shaking (120 revolutions per minute). Following incubation, lenses were washed in PBS three times to remove loosely adhered bacterial cells and transferred to tube containing 2 mL PBS and a small magnetic stirring bar and stirred for 1 min. The resulting slurry was diluted (1/10) and known volumes plated on tryptone soya agar (TSA; Oxoid). After incubation at 37 °C for 18 h, the number of CFU were calculated. Each experiment was repeated in triplicate.

### 2.7. X-ray photoelectron spectroscopy

The chemical elemental composition (atomic percentage) of the surface-coated species on the contact lenses was analysed using X-ray photoelectron spectroscopy (XPS; ESCALAB220-iXL, VG Scientific, West Sussex, England, UK). The lenses were first air dried at ambient temperature. The X-ray source used was monochromated Al Kα, with photoenergy of 1486.6 eV and source power of 120 W. The vacuum pressure was set at 10 to 8 mbar or lower. The elemental composition of the lens surfaces was determined by analysing the kinetic energy at which electrons escape the surface. XPS provides information about elemental composition and chemical and electronic state of the atoms within 10 nm depth of the surface. Changes in the atomic concentration can be interpreted as evidence of changes to the surface of the contact lenses. All surfaces measurements were conducted twice for consistency (n=2).

### 2.8. Surface charge (zeta potential)

The charge of the lenses was measured using a zetasizer (SurPASS^™^3 Antaan Pawar, Graz, Austria, Europe) with 10 mM KCI as the testing solution. The instrument was calibrated in acidic and alkaline pH. The lenses were washed in PBS and placed in a sample cell that was free from air bubbles. The measurement involved applying an electric field to the sample and measuring electrophoretic mobility through 10–20 zeta cycles. All strategies were performed in duplicate for validation (n = 3).

### 2.9. Surface hydrophobicity

The surface hydrophobicity of the contact lenses was measured using a contact angle goniometer (Ossila BV, Biopartner, 2333 BD Leiden, Netherlands) following the sessile drop technique. Contact lenses were air-dried and placed on a microscope slide mounted on a goniometer stage. Deionized water was carefully applied to the lens surface, avoiding air bubbles or excessive spreading. To measure the angle, the volume of water was increased without altering the solid-liquid interfacial area. Repeat measurements were conducted five times using different droplets and surface locations for accuracy. High-speed imaging (3–5 images) and video recording were employed to monitor changes in the contact angle over time. Software image analysis was performed using manual selection of points and using automated algorithms provided by the manufacturer (Ossila BV).

### 2.10. Contact lens parameters

The contact lens diameter was determined in accordance with International Organization of Standardisation (1836)9–3, 9338 (International Organisation for Standardization, 2012) and American National Standards Institute Z80.20–1998 protocols using a Nikon profile projector (Nippon Kogaku K. K., Tokyo, Japan) equipped with a horizontal x-y table and digital grid screen. The lens diameter was measured in a wet cell. The centre thickness was measured using Heidenhain soft contact lens thickness gauze following International Organization for Standardization 18369–3 and 9339–2 and American National Standards Institute Z80.20–1998 protocols. Five independent measurements were recorded for each lens and subsequently averaged.

### 2.11. The effect of moist thermal sterilization on activity of contact lenses

As contact lenses are usually sterilised by autoclaving during manufacture, the influence of autoclaving on the activity of lenses was investigated. After coating, the lenses were sterilised at 121 °C for 15 min in PBS. After sterilization, the ability of the lenses to reduce the adhesion of *P. aeruginosa* 6294 was determined, as described above. The experiments were repeated twice for validation.

### 2.12. Cytotoxicity of the contact lenses

*In vitro* cytotoxicity of peptidomimetic-coated lenses was determined following a direct contact method as outlined in ISO 10993–5:2009. Immortalised human corneal epithelial cells (HCE-T), transformed with an SV40 adeno vector, were used (Araki-Sasaki et al., 1995). The cells were cultured in Dulbecco’s Modified Eagle’s Medium (DMEM)/Nutrient Mixture F-12 Ham (Life Technologies, Sydney, Australia) supplemented with 10% foetal bovine serum (FBS; 10%v/v; Gibco, Sydney, Australia), 10 ng/mL recombinant human epidermal growth factor (10 ng/mL; Gibco), and 1% of a mixture of recombinant human insulin, human transferrin, and sodium selenite (Sigma-Aldrich, Sydney, Australia). Briefly, HCE-T were seeded at a density of 1 × 10^4^ cells in wells of a 24-well plate and incubated for 24–48 h at 37 °C in 5% CO_2_ to obtain an approximately 80% confluent monolayer. The coated and control uncoated lenses were transferred into these wells and incubated under the same conditions for 24 h. After incubation, the lenses were removed, 0.5 mg mL^−1^of 3–4,5-dimethylthiazol-2-yl)-2,5-diphenyl tetrazolium bromide (MTT) was applied to each well and incubated for 4 h at 37 °C in 5% CO_2_. After incubation, formazan crystals were solubilized in dimethyl sulfoxide (DMSO) and the absorbance was measured at OD570nm and compared to cells grown in the absence of lenses.

### 2.13. Data analyses

The statistical analyses were performed using GraphPad Prism version 9.5.1 (733) software (GraphPad Software, La Jolla, CA, USA). Comparisons were made using one-way ANOVA and two-way ANOVA (analyses of variance) with a Tukey’s test of multiple comparisons. The statistical significance threshold was set at *P* < 0.05.

## 3. Results

### 3.1. Optimisation of the concentration of the compounds to produce active lenses

**Fig. 2.**
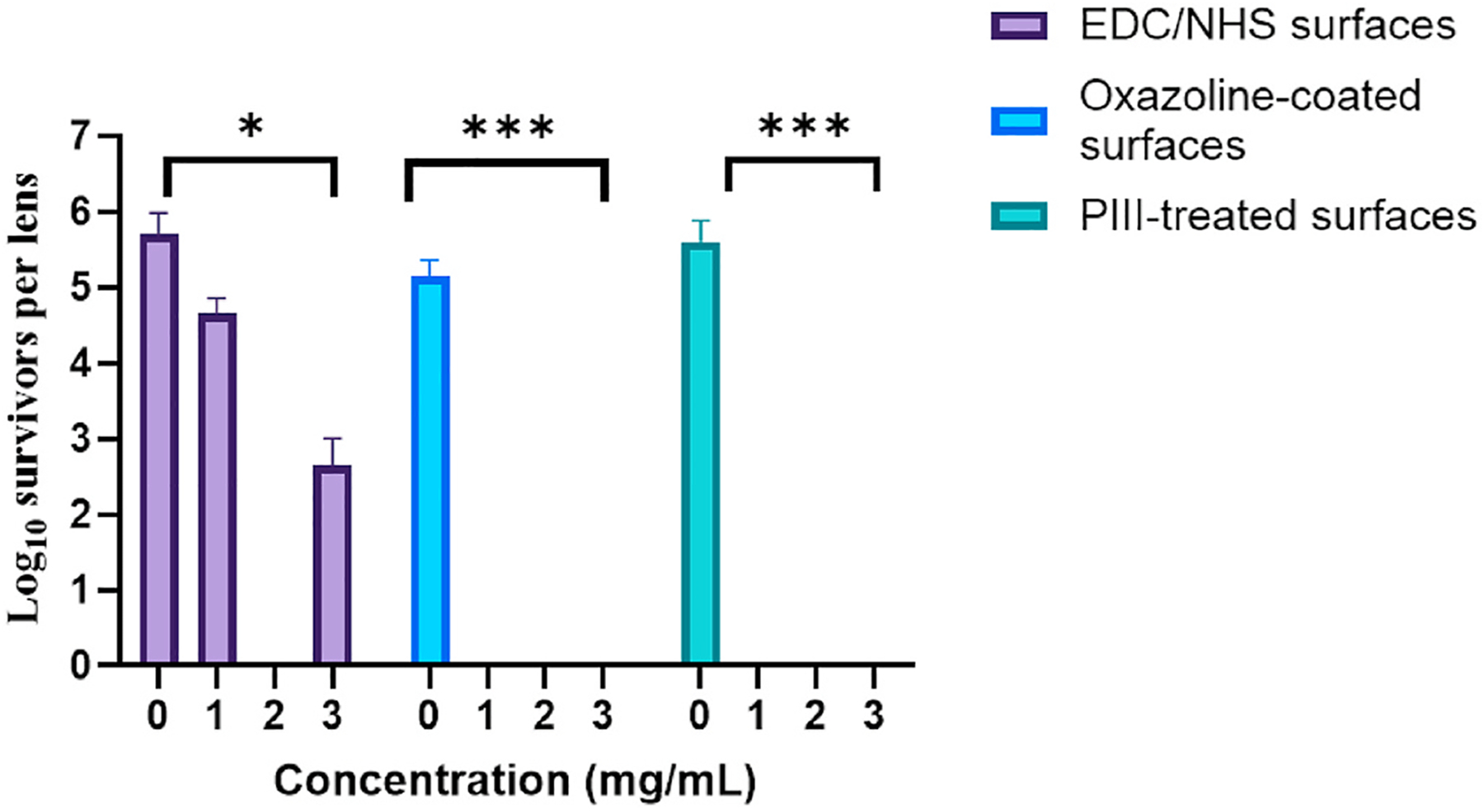
The determination of the optimal concentration of melimine for the three attachment methods to reduce the adhesion of *P. aeruginosa* ATCC 19660.*Significant difference (*p* < 0.05); ***Significant difference (*p* < 0.001). Values shown are mean and standard deviations (n = 3).

Fig. 2 shows the activity *P. aeruginosa* ATCC 19660 of contact lenses soaked in the different concentrations of melimine with all three activation methods. There was no significant difference in adhesion of *P. aeruginosa* ATCC 19660 to any of the control lenses, i.e. carbodiimide-, oxazoline- or PIII-treated surfaces alone indicating that the surface activation methods did not affect the adhesion of the bacteria. Melimine soaked at 3 mg mL^−1^ with carbodiimide activated lenses produced a reduction in numbers of viable *P. aeruginosa* of 2.4 log_10_ CFU, which was similar to previous reports with other strains of *P. aeruginosa* (Dutta et al., 2013). When melimine at 500 μg mL^−1^ was soaked with oxazoline-coated lenses, the reduction in adhesion of *P. aeruginosa* ATCC 19660 was 2.9 log_10_ CFU. When soaked at concentrations of 1 mg mL^−1^ or higher with oxazoline or PIII-treated lenses, no viable cells of *P. aeruginosa* ATCC 19660 were recovered (i.e. ≥5 log_10_ reduction in CFU).

**Fig. 3.**
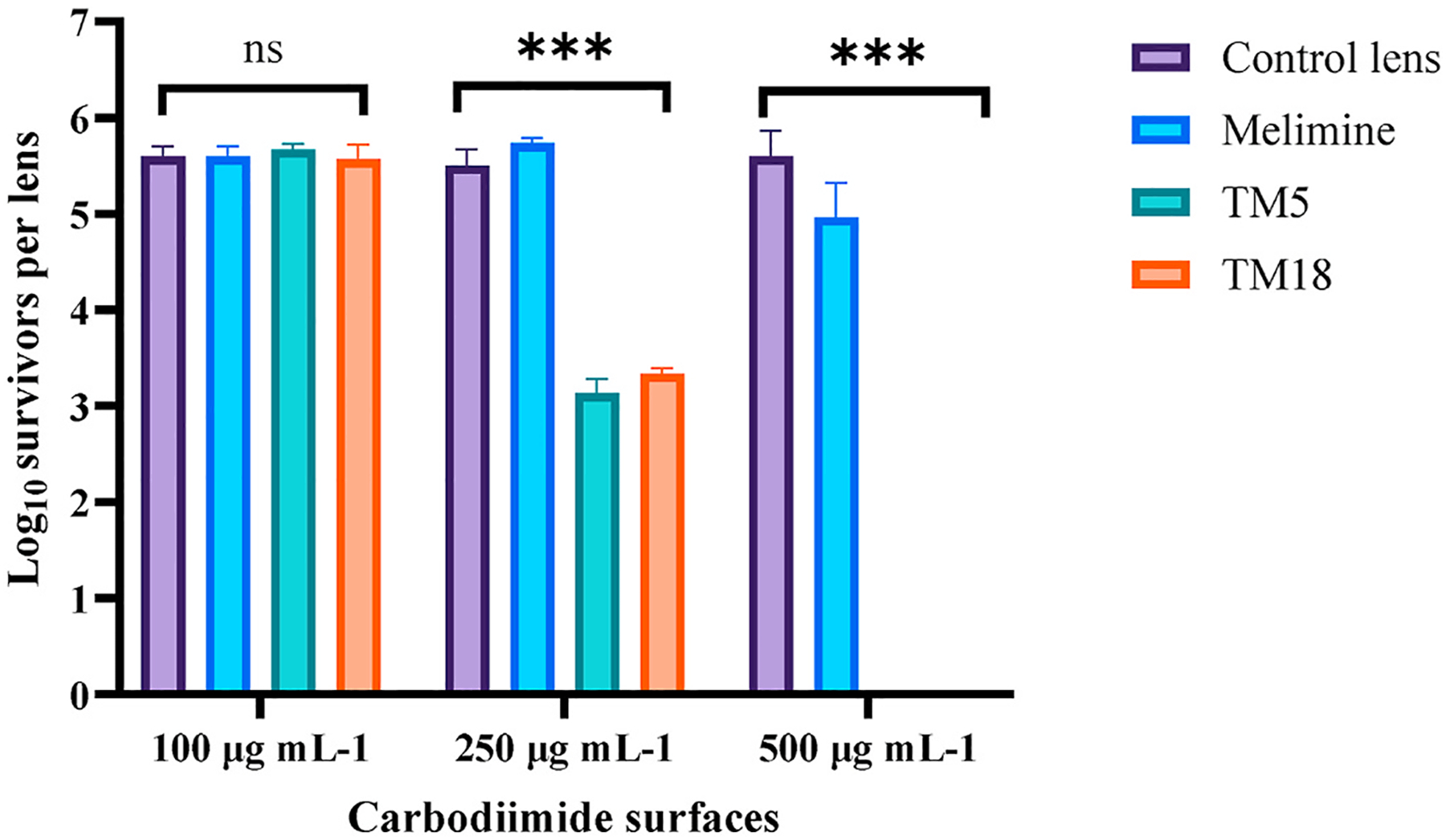
The determination of the optimal concentration of peptoids on carbodiimide surfaces. ***Significant difference (*p* < 0.001). Values shown are mean and standard deviations (n = 3).

When different concentrations of the peptoids TM5 and TM18 were allowed to covalently bind to carbodiimide activated lenses (Fig. 3), there was a dose-response, with the soaking concentration of 500 μg mL^−1^ resulting in no viable cells of *P. aeruginosa* ATCC 19660 being recovered.

**Fig. 4.**
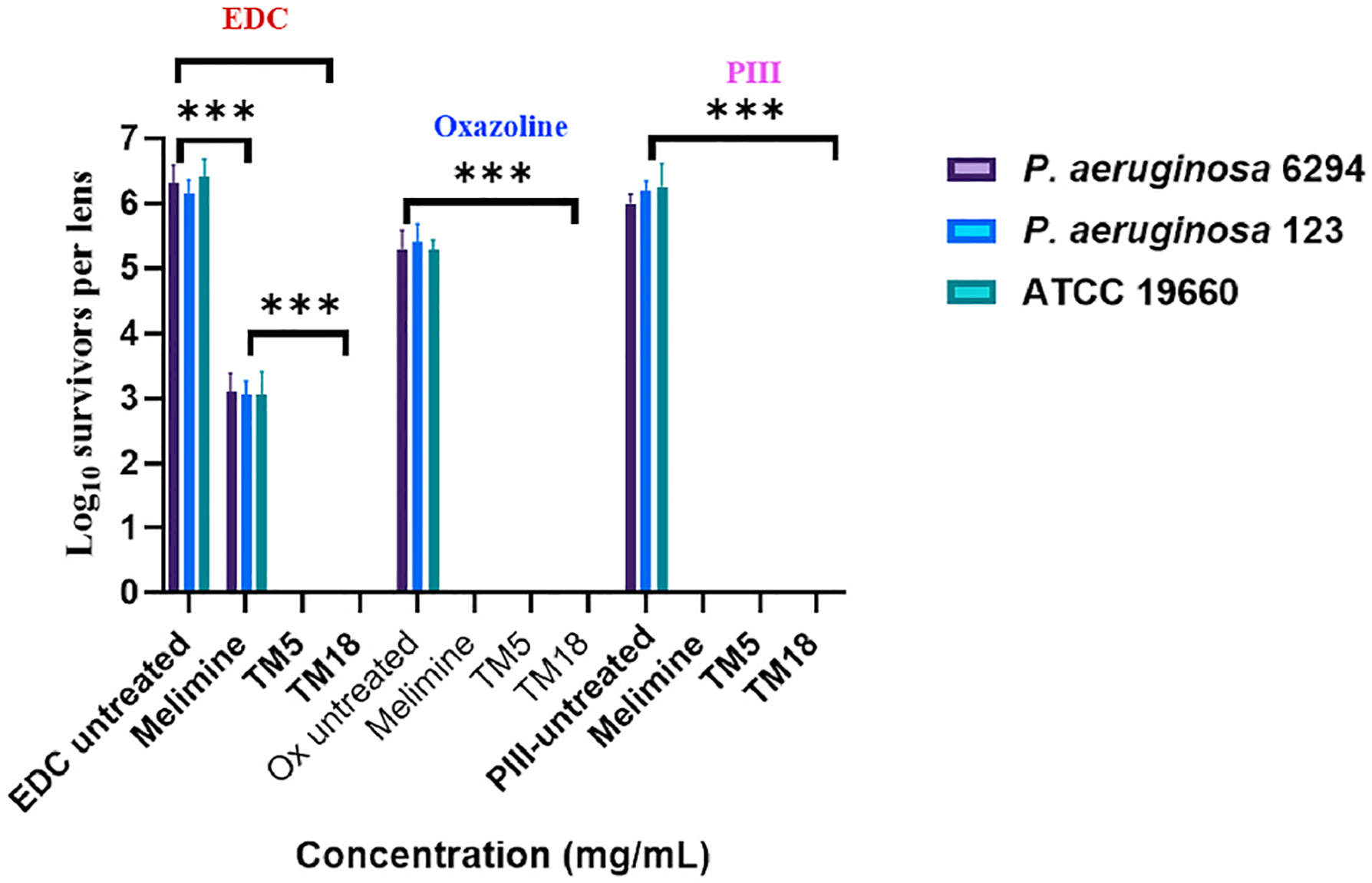
The effect of different covalent attachment strategies with melimine or peptoids to reduce the adhesion of *P. aeruginosa* strains to contact lenses. ***Significant difference (*p* < 0.001). Values shown are mean and standard deviations (n = 3).

The results for the other two strains of *P. aeruginosa*, PA123 and 6294, were similar to ATCC 19660 (Fig. 4). Carbodiimide-activated lenses soaked in 3 mg mL^−1^ melimine resulted in reductions for PA123 of 3.1 log_10_ CFU/lens and for 6294 of 3.25 log_10_ CFU/lens. Carbodiimide oxazoline or PIII-treated activated lenses soaked in 500 μg mL^−1^ TM5 or TM18 resulted in no growth for any of the three strains.

### 3.2. Elemental characterization of the coated lenses

In order to determine whether the compounds had been bound to the contact lens surface, changes to the elemental composition of the lens surfaces were investigated using XPS. Following treatment with carbodiimide and subsequent binding of melimine, TM5, or TM18 to the lenses, noticeable differences in elemental composition were detected compared to the control sample (Table 1). The most notable changes were observed in the nitrogen percentage (N%), which increased by 542% for melimine, 225% for TM5, and 192% for TM18.

When lenses were treated with oxazoline only, compared to the untreated lenses, there was 11% reduction in C%, and a 58% increase in N%. After allowing melimine to bind to the lenses there were only relatively small changes in elemental compositions. On the other hand, the most noticeable changes for TM5 and TM18 when bound to the oxazoline-deposited lenses were reduction of N% to 51% and 58%, respectively.

When lenses were treated with PIII only, compared to the untreated lenses, there was a 16% reduction in C%, 4% increase in O% and a 520% increase in N%. After allowing melimine to bind to the lenses, there were relatively small changes in elemental composition. Similarly to the oxazoline plasma deposited lenses, the most noticeable changes after TM5 or TM18 bound to the PIII-treated lenses decreases N% of were 61% and 60% respectively.

### 3.3. The effect of coating on the charge of the contact lenses

**Fig. 5.**
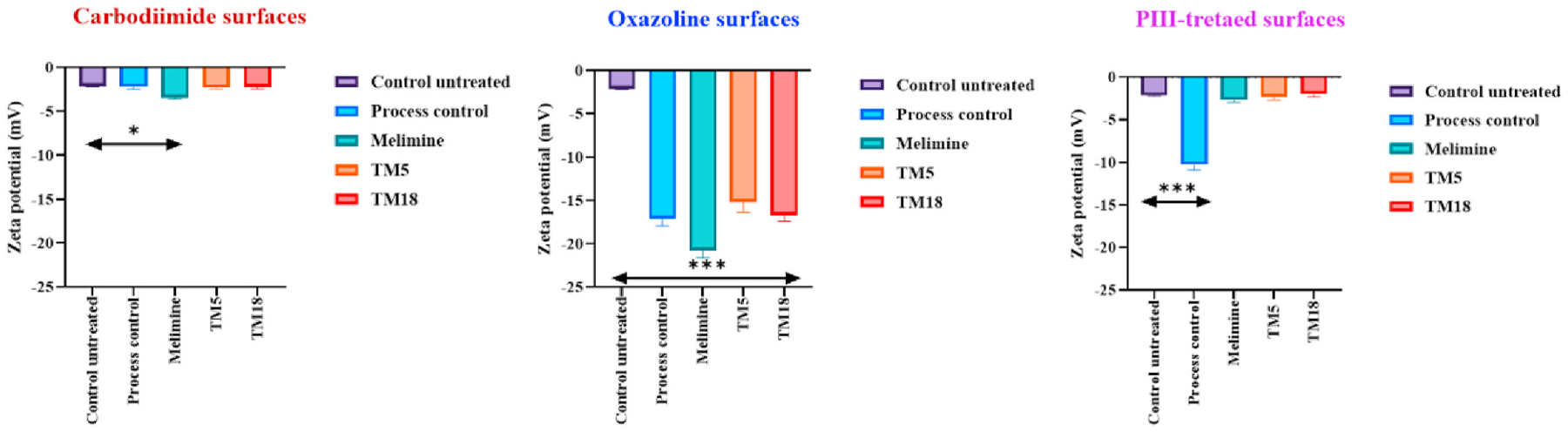
The charge of antimicrobial contact lenses functionalised via different strategies.*Significant difference (*p* < 0.05); ***Significant difference (*p* < 0.001). Values shown are mean and standard deviations (n = 3).

The charge of surfaces can affect bacterial adhesion (Zheng et al., 2021), therefore changes in charge due to the addition of the cationic melimine and peptoids was determined. TM5 or TM18 attached to contact lenses activated by carbodiimide did not significantly (*p* ≥ 0.94) change the charge of the etafilcon A contact lenses, whereas the attachment of melimine resulted in a small but significant increase in the negativity (*p* < 0.0001) (Fig. 5). On the other hand, oxazoline deposition of the etafilcon A lenses resulted in a significant increase in negativity of the surface (*p* < 0.0001). The addition of melimine or TM5 to the oxazoline-deposited lenses significantly changed the zeta potential (*p* < 0.0001), whereas the addition of TM18 did not alter the zeta potential (Fig. 5) compared to that of oxazoline alone. Activation of the etafilcon A lenses with PIII significantly increased the negativity of the lens surface (*p* < 0.0001) (Fig. 5), and addition of any of the three compounds significantly decreased the surface negativity compared to PIII-treatment alone (*p* < 0.0001), but there were no differences between the compounds.

### 3.4. Hydrophobicity of the coated lenses

The hydrophobicity of a contact lens surface is a measure of its wettability, with hydrophobic surfaces wetting more poorly than hydrophilic surfaces, and this can modulate bacterial adhesion (Vijay et al., 2012). The wettability of the contact lens surfaces after coating using the three different techniques is given in (Table 2). Overall, there were slight to moderate increases in hydrophobicity (contact angle) after addition of the peptoids, regardless of the chemistry used, compared to uncoated control lenses.

### 3.5. Contact lens parameters

Contact lens parameters such as diameter and lens centre thickness on all three surfaces did not show any significant difference from control lenses (Table 3).

### 3.6. The effect of autoclaving on activity of contact lenses

**Fig. 6.**
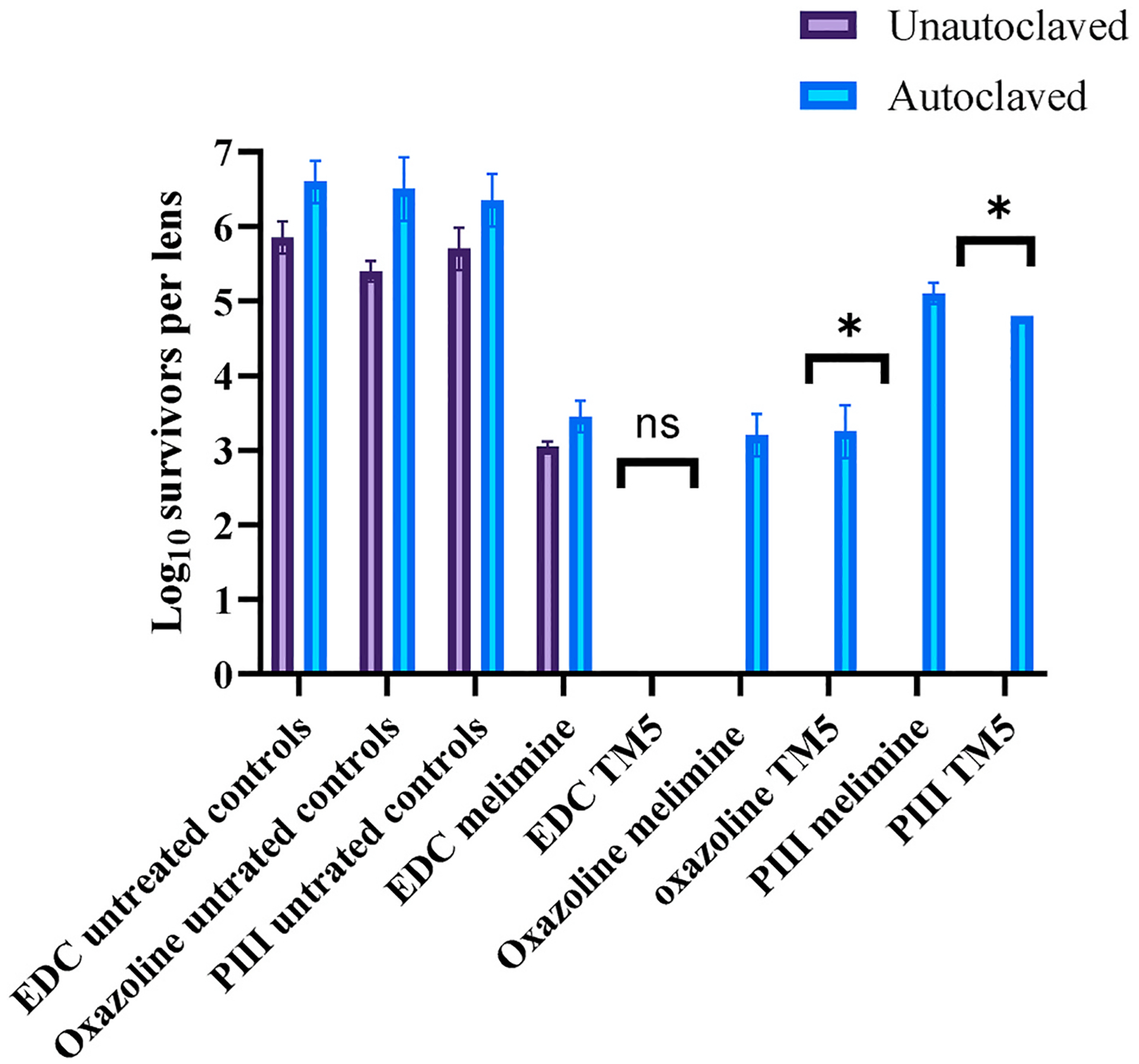
The effect of autoclaving on the antimicrobial activity of melimine and TM5 coated via different strategies.*Significant difference (*p* < 0.05). Values shown are mean and standard deviations (n = 2).

Prior research had demonstrated that melimine, when bound to etafilcon A contact lenses via carbodiimide chemistry, retained the majority of its activity towards *P. aeruginosa* 6294 after autoclaving (Dutta et al., 2013, 2016). In the current study, the activity of TM5 was also retained after autoclaving with carbodiimide-activated lenses (Fig. 6). Autoclaving did not alter the activity of melimine bound via oxazoline, but significantly (*p* = 0.01) reduced the activity of TM5 bound via oxazoline, and melimine or TM5 bound via PIII.

### 3.7. Cytotoxicity of coated lenses

Due to the loss of activity with the oxazoline and PIII-treated lenses after autoclaving, only carbodiimide activated contact lenses were tested for their cytotoxicity and compared to untreated lenses. Melimine and TM18, when bound to etafilcon A contact lenses, resulted in 21 ± 5% and 28 ± 4% cytotoxicity, respectively, to human corneal epithelial cells. In contrast, no cytotoxicity was observed with TM5 coated lenses. The toxicity of TM5 was significantly less from that of TM18 and cationic melimine (*p*-0.001). All three compounds exhibited cytotoxicity levels below the established safety threshold of 30% cell death (International Organization for Standardization, 2009).

## 4. Discussion

This study focused on comparing three attachment strategies for the antimicrobial peptide melimine and the peptoids TM5 and TM18 on etafilcon A contact lenses. Etafilcon A was chosen due to its widespread use, accounting for approximately 50% of contact lenses used by wearers worldwide (Morgan et al., 2024). Its high-water content, ionic nature, and established safety and comfort profile (Efron et al., 2020) make it a relevant and clinically significant. It has also been used in laboratory and clinical trials of melimine- and Mel4-coated lenses and so allowed direct comparisons with those previous results (Dutta et al., 2013, 2014, 2016, 2017; Kalaiselvan et al., 2021, 2022). This study found that binding the peptoids TM5 and TM18 as well as the peptide melimine to lenses caused changes in the lens surface elemental composition, wettability and surface charge. Whilst surface charge and wettability can modulate bacterial adhesion (Vijay et al., 2012; Zheng et al., 2021), the results suggest these had little overall affect as adhesion was not correlated to these surface changes and the changes caused by the surface activation methods did not affect the adhesion of the bacteria. The adhesion was reduced most likely by the activity of the peptoids and melimine.

The study confirmed previous findings that melimine, covalently bound to etafilcon A lenses via carbodiimide chemistry, significantly reduced the adhesion of *P. aeruginosa* strains by ≥ 2 log10 CFU per lens (Dutta et al., 2013). Additionally, the current study demonstrated that melimine attached via oxazoline or plasma immersion ion implantation (PIII) strategies provided even greater inhibition of *P. aeruginosa* adhesion, even with a lower concentration of melimine. Notably, the reduction in adhesion with melimine on oxazoline-treated surfaces remained stable after autoclaving, while PIII-treated surfaces did not retain their stability post-autoclaving. Previously, PIII had been used to bind melimine to polyvinyl chloride surfaces, achieving a reduction in *Staphylococcus aureus* adhesion by approximately 2 log10 CFU (Tran et al., 2019a). These results suggest that oxazoline-treated etafilcon A lenses offer a promising and durable approach for reducing bacterial colonization, particularly in daily disposable contact lenses, where the risk of contamination is high importantly due to either misuse.

The activity of the peptoids could be predicted from their antimicrobial activity in solution. The peptoids TM5 and TM18, which had previously been shown to be highly active against *P. aeruginosa* in solution (Sara et al., 2024), were also highly active when attached to etafilcon A contact lenses via any of the three methods, resulting in ≥5.4 log_10_ CFU per lens reduction in adhesion of all three strains of *P. aeruginosa*. Similar findings have been observed on titanium oxide modified surfaces using similar peptoids (Statz et al., 2008). One of these previous publications showed that activity of bound peptoids was at least partially predicted on the activity in solution, similar to the current study where TM5 and TM18 had previously been shown to have relatively low MICs to *P. aeruginosa* strains in solution (Sara et al., 2024). However, whilst covalently binding TM5 via carbodiimide chemistry remained stable after autoclaving, similar to the previous findings with melimine-coated lenses, binding via oxazoline or PIII lost activity after autoclaving. The loss of activity for oxazoline may be due to the susceptibility of the types of bonds it forms.

The oxazoline-plasma process results in the formation of oxazoline rings, isocyanates, alkynes, and nitriles on surfaces, which can interact with arginine or lysine amino acids in AMPs or peptoids to produce intermediate imidazole derivatives (Macgregor-Ramiasa et al., 2015; Suda et al., 2016; Zanini et al., 2016). This subsequently results in the formation of amide or ester bonds. Amide bonds are thermally stable, but ester bonds are susceptible to hydrolysis at high temperatures (Lee et al., 2017). The current study suggests that melimine reacted with the oxazoline surface to produce, at least in part, thermally stable bonds, which were probably amides. TM5, lacking carboxylic acids, may partially react with isocyanates and other electrophiles present in oxazoline plasma. This reaction results in the formation of both thermally stable and unstable bonds, thereby retaining partial activity of ≥2log10 inhibition (Mees et al., 2016; Suda et al., 2016). On the other hand, PIII surfaces enable covalent attachment of wide range of molecules (Tran et al., 2019b). As most contact lenses are terminally sterilised by autoclaving prior to release from the manufacturer, oxazoline or PIII, as they are used in the current protocols, are not appropriate methods for producing antimicrobial contact lenses with peptoids, but the use of carbodiimide, which attaches AMPs and peptoids via heat stable amide bonds is an attractive option. The radical concentration in PIII treated surfaces decreases over time and is affected by the temperature of heat treatments which accelerate the diffusion of radicals. There is a possibility that a more intense PIII treatment could be delivered closer to the time of autoclaving that would enable an improved retention of the action, since retention of Mel4 activity after autoclaving has been found on PIII treated PEEK and PEK surfaces (Kruse et al., 2024).

The current study showed that covalent attachment of melimine or the peptoids to the lenses resulted in changes to the hydrophobicity and charge of the lenses. These assays were conducted to assess how the modifications of the contact lenses, specifically the attachment of peptoids, altered surface properties that are important not only as they can affect bacterial adhesion but also because they can affect the biocompatibility of contact lenses during wear. A hydrophilic, i.e. wettable, lens surface may help improve biocompatibility (Willcox et al., 2021), for example by reducing lipid deposition. Charged surfaces may stimulate interactions with tear film proteins or disinfectants (Willcox et al., 2021). Elemental characterization via XPS provides confirmation of successful peptoid binding. In the case of 2-methyl, 2-oxazoline, nitrogen is present in its five-membered oxazoline ring. When this polymer is grafted or deposited onto a surface, the nitrogen atoms from the oxazoline ring become part of the surface chemistry. Subsequent attachment of melimine, which contains 74 nitrogen atoms maintains a high nitrogen content on the surface. The reduction of N% after the addition of TM5, which contains 8 nitrogen atoms, or TM18, which contains 12 nitrogen atoms is likely due to the coverage of the underlying oxazoline by these compounds.

A previous study has shown that adhesion of *P. aeruginosa* to hydrogel contact lenses including etafilcon A can be affected by the hydrophobicity of the lens, with a more hydrophobic surface resulting in lower adhesion (Vijay et al., 2012). The slight increased hydrophobicity observed after covalently attaching melimine and peptoids, particularly TM5, could be attributed to the activation of functional groups on their surfaces. However, the large decrease in adhesion is unlikely solely to be the result of only the relatively small change in hydrophobicity. These findings indicate that the direct action of melimine and the peptoids on the bacteria has the most pronounced effect, which is probably mediated via their positive charges interacting with the outer surfaces of *P. aeruginosa* (Chongsiriwatana et al., 2008; Yasir et al., 2019).

## 5. Conclusion

The findings of this study offer promising insights into the development of antimicrobial contact lenses. The study demonstrates that the proteolytically stable peptoids TM5 and TM18 (Sara et al., 2024) when bound via carbodiimide chemistry, it can significantly reduce the adhesion of *P. aeruginosa* to contact lenses and provide a thermally stable active surface. Notably, TM5 showed significantly lower cytotoxicity compared to TM18 and the cationic melimine. While further research is needed to fully explore the clinical applications of these findings, the study provides a strong foundation for the development of more effective and infection-resistant contact lenses. In addition, the novel attachment strategies developed in this study for peptoids TM5 and TM18, as well as cationic melimine, could be applied to other polymer surfaces beyond contact lenses. This broader application potential highlights the versatility and scalability of these techniques, paving the way for the development of a wide range of medical devices and materials with enhanced antimicrobial properties.

## 6. Limitations

While this study presents promising insights into the development of antimicrobial contact lenses, there are some limitations. These include the unexplored long-term stability of peptoid surfaces and the study’s focus on a specific bacteria, even though *P. aeruginosa* is the most common cause of microbial keratitis associated with contact lens wear (Ahanotu and Ahearn, 2002; Prabha et al., 2007). These aspects should be studied in future research. Other future studies should examine the durability of the surface coating to repeated washing and the use of cleaning and disinfecting regimens. The long-term effects, potential cytotoxicity on human cells, and the risk of bacterial resistance were not extensively explored. Further research is needed to confirm these findings *in vivo* and to assess the broader applicability and safety of these antimicrobial strategies in various medical contexts.

## Figures and Tables

**Table 1:** XPS analysis of antimicrobial contact lenses (mean ± SD; N = 2).

Elemental composition					
Lenses	Carbon%	Oxygen%	Nitrogen%	C/O	C/N
**Carbodiimide activation**					
Control untreated	70 ± 4.8	25 ± 5.4	0.9 ± 0.8	2.8	0.01
Process control	66 ± 0.5	30 ± 0.6	1.2 ± 0.9	2.2	0.01
Melimine	70 ± 5.9	19 ± 3.7	7.7 ± 3.5	3.7	0.1
TM5	74.2^a^	18.8^a^	3.9^a^	3.9	0.05
TM18	70 ± 3.7	22 ± 3.0	3.5 ± 0.1	3.0	0.05
**Oxazoline activation**					
Control untreated	71 ± 0.0	23 ± 0.5	1.2 ± 0.7	3	0.02
Process control	63 ± 4.1	23 ± 2.3	7.9 ± 0.1	2.7	0.12
Melimine	62 ± 3.0	22 ± 0.9	7.7 ± 0.1	2.8	0.12
TM5	54 ± 1.9	26 ± 6.8	3.9 ± 1.0	2.0	0.07
TM18	52 ± 2.4	26 ± 0.3	3.3 ± 0.0	1.9	0.06
**PIII-treatment**					
Control untreated	71 ± 0.0	23 ± 0.4	1.5 ± 0.1	3	0.02
Process control	60 ± 0.0	24 ± 0.4	9.3 ± 0.1	2.4	0.15
Melimine	56 ± 4.8	28 ± 1.0	8.1 ± 0.9	2.0	0.14
TM5	53 ± 3.3	26 ± 0.7	3.6 ± 0.4	2.0	0.06
TM18	63 ± 3.4	24 ± 0.1	3.7 ± 0.6	2.5	0.05

a One replicate analysed.

**Table 2:** Antimicrobial-bound lens surfaces contact angle (o) (mean ± SD; N = 5).

Contact lenses	Carbodiimide	Oxazoline	Plasma
Control uncoated	29 ± 0.2	29 ± 0.2	29 ± 0.2
Process control	33 ± 0.0	74 ± 1.4	55 ± 0.5
Melimine	38 ± 1.0	39 ± 2.0	29 ± 0.2
TM5	44 ± 0.9	40 ± 0.6	52 ± 0.1
TM18	38 ± 0.9	27 ± 2.8	44 ± 0.1

**Table 3:** Changes in contact lens parameters after activation and addition of the antimicrobials (mean ± SD; N = 5).

Mean ± SD+	Carbodiimide surfaces		Oxazoline surfaces		PIII-coated surfaces	
	Diameter	Centre thickness	Diameter	Centre thickness	Diameter	Centre thickness
Control uncoated	14.2 ± 0.01	0.085 ± 0.00	14.0 ± 0.01	0.084 ± 0.00	14.1 ± 0.02	0.084 ± 0.00
Process control	14.2 ± 0.01	0.086 ± 0.00	14.1 ± 0.01	0.089 ± 0.00	14.1 ± 0.01	0.088 ± 0.00
Melimine	14.2 ± 0.01	0.091 ± 0.00	14.1 ± 0.00	0.087 ± 0.00	14.1 ± 0.01	0.087 ± 0.00
TM 5	14.2 ± 0.01	0.088 ± 0.00	14.1 ± 0.00	0.086 ± 0.00	14.1 ± 0.01	0.087 ± 0.00
TM 18	14.2 ± 0.01	0.087 ± 0.00	14.1 ± 0.01	0.087 ± 0.00	14.1 ± 0.01	0.084 ± 0.00

## Data Availability

Data will be made available on request.
